# Glycyrrhizic Acid Inhibits SARS-CoV-2 Infection by Blocking Spike Protein-Mediated Cell Attachment

**DOI:** 10.3390/molecules26206090

**Published:** 2021-10-09

**Authors:** Jingjing Li, Dongge Xu, Lingling Wang, Mengyu Zhang, Guohai Zhang, Erguang Li, Susu He

**Affiliations:** 1 State Key Laboratory of Pharmaceutical Biotechnology, Medical School, Nanjing University, Nanjing 210093, China; lijingjing@topcelbio.com (J.L.); 13337718237@163.com (D.X.); wanglinling1010@163.com (L.W.); mg1935020@smail.nju.edu.cn (M.Z.); 2 Jiangsu Key Laboratory of Molecular Medicine, Medical School, Nanjing University, Nanjing 210093, China; 3 Jiangsu Topcel Biological Technology Co., Ltd., Nanjing 210093, China; 4 Yancheng Medical Research Centre, Medical School, Nanjing University, Yancheng 224000, China; 5 Institute of Medical Virology, Nanjing Drum Tower Hospital, Medical School, Nanjing University, Nanjing 210093, China; 6 State Key Laboratory for Chemistry and Molecular Engineering of Medicinal Resources, School of Chemistry and Pharmaceutical Sciences, Guangxi Normal University, Guilin 541006, China; zgh1207@gxnu.edu.cn; 7 Shenzhen Institute of Nanjing University, Shenzhen 518000, China

**Keywords:** glycyrrhizin, SARS-CoV-2, surface plasmon resonance, autodocking

## Abstract

Glycyrrhizic acid (GA), also known as glycyrrhizin, is a triterpene glycoside isolated from plants of *Glycyrrhiza* species (licorice). GA possesses a wide range of pharmacological and antiviral activities against enveloped viruses including severe acute respiratory syndrome (SARS) virus. Since the S protein (S) mediates SARS coronavirus 2 (SARS-CoV-2) cell attachment and cell entry, we assayed the GA effect on SARS-CoV-2 infection using an S protein-pseudotyped lentivirus (Lenti-S). GA treatment dose-dependently blocked Lenti-S infection. We showed that incubation of Lenti-S virus, but not the host cells with GA prior to the infection, reduced Lenti-S infection, indicating that GA targeted the virus for infection. Surface plasmon resonance measurement showed that GA interacted with a recombinant S protein and blocked S protein binding to host cells. Autodocking analysis revealed that the S protein has several GA-binding pockets including one at the interaction interface to the receptor angiotensin-converting enzyme 2 (ACE2) and another at the inner side of the receptor-binding domain (RBD) which might impact the close-to-open conformation change of the S protein required for ACE2 interaction. In addition to identifying GA antiviral activity against SARS-CoV-2, the study linked GA antiviral activity to its effect on virus cell binding.

## 1. Introduction

The uncertainty of the coronavirus disease 2019 (COVID-19) pandemic situation remains elusive, although great success has been accomplished with the rapid development of protective vaccines against severe acute respiratory syndrome coronavirus 2 (SARS-CoV-2). There are currently no effective drugs for the treatment of COVID-19, although clinical trials and case reports involving antiviral and antiparasitic agents have yielded promising results that warrant further investigation [1,2]. Herbal medicines have a long history of vindicated clinical efficacy against infectious diseases. Clinical evidence shows that herbal medicines are effective against viral infections such as influenza, SARS, and SARS-CoV-2 by targeting virus cell entry, viral replication, and host antiviral immune response steps. During the pandemic of COVID-19, several drugs formulated according to principles of Chinese medicine showed therapeutic effects against mild and severe COVID-19. Among the drugs and formulae recommended by the Chinese authority for COVID-19 therapy, the dried root of *Glycyrrhiza* spp. (licorice) is among the most commonly used ingredients in the formulae [3]. The radices of *Glycyrrhiza* spp. have been used as an important ingredient of herbal medicines and also a flavoring agent in traditional formulae since antiquity [4,5]. The use of the dried roots of the plant can be traced back to ancient Assyrian, Egyptian, Chinese, and Indian cultures for symptoms that resemble those of viral respiratory tract infections such as dry cough or hoarse voice [5,6]. Recent reports also suggest that licorice extract may have a potential role in combating COVID-19 and associated conditions [7].

The main chemical component from *Glycyrrhiza* sp. is glycyrrhizic acid (GA), also known as glycyrrhizin, a triterpenoid saponin that can be extracted from the dried roots in high yields [8,9]. Numerous reports show that GA is effective against the infection of enveloped and nonenveloped viruses such as herpes viruses, respiratory syncytial virus, vaccinia virus, and SARS-CoV in cell culture studies [4,10,11]. Cinatl and colleagues found that glycyrrhizin was among the most active compounds tested in inhibiting replication of the SARS-CoV [12]. Thus, licorice and GA could be an old weapon against emerging diseases [13,14].

In this study, we investigated the antiviral effect of GA against SARS-CoV-2 using a pseudotyped lentivirus that has the SARS-CoV-2 S protein on its envelope (Lenti-S). We found that GA inhibited Lenti-S infection through inhibition of virus attachment to host cells. Preincubation of Lenti-S, rather than the host cells, with GA prior to the infection reduced Lenti-S infection, suggesting that GA primarily targets the virus rather than the host cells. GA interacted with the S protein with high affinity and blocked a recombinant S protein binding to the host cells. Thus, this study uncovered a mechanism by which GA blocks SARS-CoV-2 infection by impeding virus and host cell interaction.

## 2. Materials and Methods

### 2.1. Cells, Reagents, and Antibodies

Vero E6 and 293T cells were obtained from Cell Bank of Chinese Academy of Sciences and were cultured in Dulbecco’s Modified Eagle Medium (DMEM) supplemented with 10% fetal bovine serum (FBS, *v*/*v*) at 37 °C in a humidified incubator. The medium also contained 10 mM HEPES (pH 7.4), 2 mM GlutaMAX, and penicillin–streptomycin–fungizone (100 units/mL of penicillin, 100 μg/mL of streptomycin, and 250 ng/mL of amphotericin B). The DMEM and all the supplemented ingredients were purchased from Invitrogen (Shanghai, China). An insect cell-expressed S protein (40589-V08B1, S1 + S2 ectodomain) and a polyclonal antibody cross-reactive to SARS-CoV-2 S (40150-T62) were purchased from Sino Biological (Beijing, China). The Steady-Lumi firefly luciferase reporter gene assay reagent was purchased from Beyotime (Nantong, China). An HIV-1 NL4-3 luciferase reporter vector that contains defective Nef, Env, and Vpr was purchased from MiaoLing (P20782, Wuhan, China). pCMV3-SARS-CoV-2-S (VG40589-UT, accession number MN908947.3) with codon-optimized *S* gene for mammalian cell expression and pCMV3-ACE2-HA (HG10108-CY) were purchased from Sino Biological. The pCMV3-SARS-CoV-2-S was modified in the lab by deletion of the last 19 amino acid residues to generate pCMV3-S since the endoplasmic reticulum (ER)-retention signal from the cytoplasmic tail was reported to interfere with virus preparation [15,16]. Glycyrrhizin ammonium salt (#50531, purity ≥ 95%) and HRP-conjugated anti-HA antibody (H3663) were purchased from Sigma-Aldrich. A stock solution of 50 mM GA was prepared by dissolving the compound in distilled water with pH adjusted to 7.4 using NaOH.

### 2.2. Spike Protein-Pseudotyped Virus (Lenti-S) Preparation

The spike protein-S-pseudotyped lentivirus (Lenti-S) was generated using a 2-plasmid system as previously reported [17]. Stocks of single-round infection of S protein-pseudotyped virus were produced by cotransfection of 293T cells (1.0 × 10^7^ cells per 10 cm dish) with 2 μg of pCMV-S plasmid and 8 μg of pNL4.3-Luc using PEI reagent. The supernatant was harvested 34 h following transfection. After clarification by centrifugation at 1500× *g* followed by 0.45 μm filtration, pseudovirus-containing medium was collected and aliquots were stored at −80 °C. The virus was validated by testing for its ability to deliver the luciferase gene to ACE2-expressing HEK293T cells [18]. 

### 2.3. Infection Assay

Pseudotyped viruses provide an efficient way to determine an antiviral effect by measuring reporter gene expression. For gene-transducing assay, the permissible Vero E6 cells in 96-well plates (#3599, Costar, Corning, NY, USA) were infected with varying amounts of Lenti-S. Luciferase expression was determined at approximately 24 h PI using Steady-Lumi reagent on a GloMax 96 luminometer (Promega, Madison, WI, USA). 

For blocking assay, Vero E6 cells were detached using 3 mM ethylenediaminetetraacetic acid (EDTA). After washing twice with serum-free medium (SFM), the cells were resuspended in ice-cold SFM-containing 2% (*v*/*v*) FBS (10^7^ cells/mL). The cells were then aliquoted (5 × 10^5^ cells/sample), and duplicate samples were incubated on ice with Lenti-S in the absence or presence of GA or a blocking reagent as indicated. The mixtures were incubated on ice for 60 min with occasional mixing. At the end, the cells were then washed with ice-cold medium 3 times to remove non-bound virus then plated in 12-well plates (Corning™ Costar, #3512) without supplementation of GA or the blocking reagent. Luciferase expression in duplicate samples was determined at 24 h PI.

### 2.4. Protein Biotinylation and Binding Assay

An insect-cell-expressed S protein (2 μg), resuspended in 100 μL of 50 mM NaHCO_3_, was labeled with freshly prepared sulfo-NHS-biotin (21217, Pierce, Carlsbad, CA, USA, 0.5 mg in 50 μL NaHCO_3_). The reaction lasted for 10 min at room temperature. At the end, unreacted sulfo-NHS-biotin was quenched by reaction with 1 mg glycine dissolved in 20 μL NaHCO_3_. The labeled protein was dialyzed against phosphate-buffered saline (MW cutoff 12 kD) and was used for protein-binding assay.

To measure the effect of GA on S protein binding, detached Vero E6 cells were incubated on ice with the biotinylated S protein in the absence or presence of varying amounts of GA or excess amounts of unlabeled S protein. After incubation on ice for 60 min, the cells were washed 3 times with ice-cold medium. After the cell lysates were separated by a 6% SDS-PAGE gel, cell-attached S protein was detected by immunoblotting analysis using an HRP-conjugated anti-biotin antibody (A0185, Sigma-Aldrich, St Louis, MO, USA). Anti-actin (sc-8432, Santa Cruz, Dallas, TX, USA) was used for loading controls.

### 2.5. Surface Plasmon Resonance (SPR) Studies

SPR analysis was performed using the Biacore T200 system and a CM5 sensor chip. The chip consists of two channels that were loaded with S protein. Briefly, the carboxymethylated dextran matrix was activated via the passage of EDC-HCl and NHS (0.2 M and 0.05 M in water, respectively). Then, the S protein at 20 μg/mL (in 1 mM sodium acetate) was passed over the surface. Any remaining un-reacted active ester groups were quenched by the passage of ethanolamine solution. The sensor chip surface was regenerated after each experiment by passing sodium dodecyl sulfate (SDS, 100 mM) over the surface for 2 min. This simple procedure reliably returned the original signal response seen before the binding experiment started. For testing, GA at concentrations was passed over the chip, and SPR angle changes were recorded and reported as response units (RUs). Data fitting was performed using the 1:1 Langmuir model in the BIAevaluation software package (GE Healthcare, Waukesha, WI, USA).

### 2.6. Autodocking

Docking analysis was performed using AutoDock Vina 1.1.2 (Windows version) with default scoring function [19]. The molecular structures of SARS-CoV-2 S protein (PDB id: 6vsb) and ACE2 (PDB id: 6m18) were downloaded from the Research Collaboratory for Structural Bioinformatics-Protein Databank (RCSB-PDB) [20]. The 3D structure of GA was from ZINC (https://zinc.docking.org/, id: 960251743495; last accessed on 20 September 2021). The structures were prepared using AutoDock Tools (ADT) by addition of polar hydrogens and the Gasteiger charges. Structures were exported in the pdbqt format after assigning the AD4 (AutoDock 4) atom type [21]. In the AutoDock Vina configuration files, the parameter num_modes was set to 1000 and exhaustiveness to 100. We chose all the rotatable bonds in ligands to be flexible during the docking procedure, and we kept all the residues of S protein inside the binding pockets rigid. Multiple rounds of definition of the grid coordinate (x-, y-, and z-coordinates) with a defined grid box size were conducted to screen through the entire extracellular part of the S protein. The resulting docking structures were further analyzed in PyMOL.

### 2.7. Statistical Analysis

The assays were performed at least 2 times independently. Data were analyzed with Excel (Microsoft) for statistical significance using Student’s *t* test. *p* < 0.05 was considered significant.

## 3. Results

### 3.1. Inhibition of GA against SARS-CoV-2 Infection against S Protein-Pseudotyped Virus

**Figure 1:**
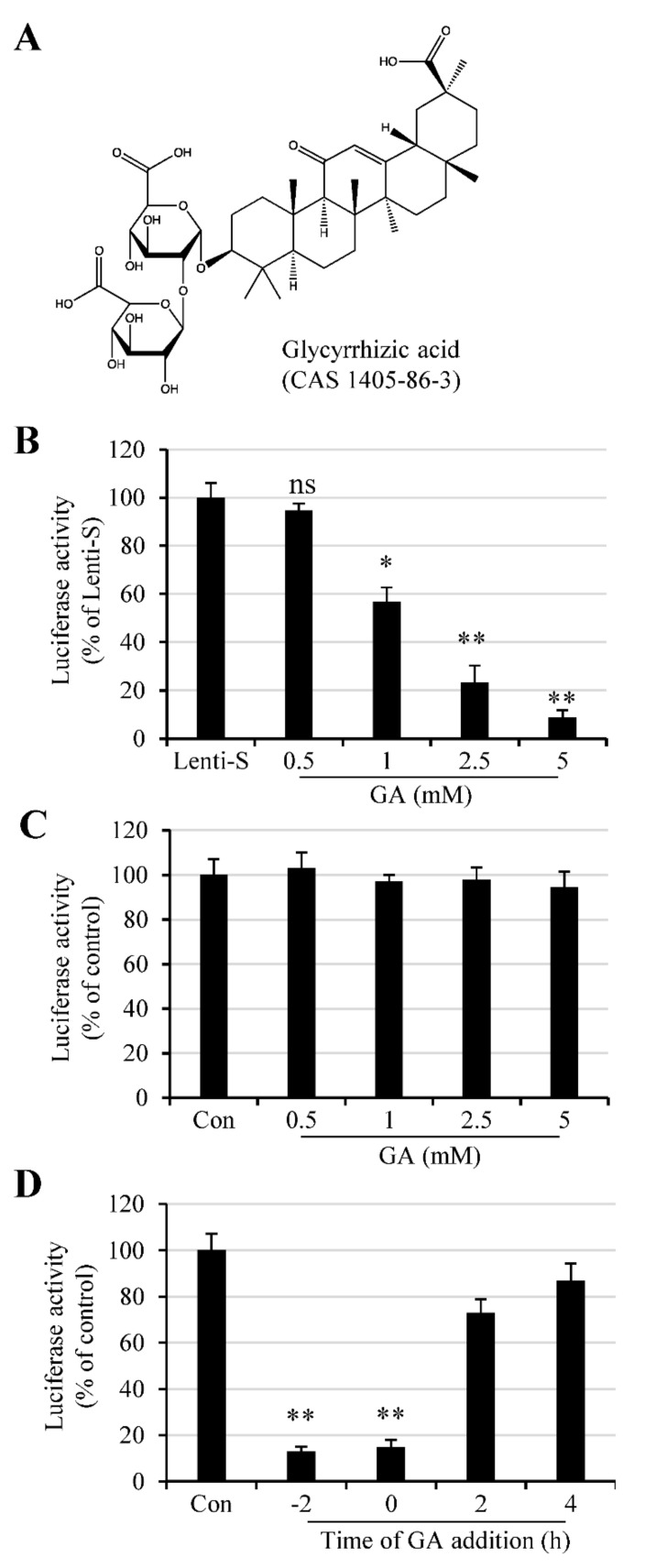
GA effect on Lenti-S infection. (**A**) Molecular structure of glycyrrhizic acid. (**B**) Effect of GA on Lenti-S infection. Vero E6 cells were infected with Lenti-S pseudovirus in the absence or presence of GA at indicated concentrations for 24 h. Luciferase activity was determined. Data are expressed as a percentage of untreated controls (Lenti-S). The experiment was performed twice, and data are mean ± SD of triplicate wells. Independent two-sample comparisons between non-GA treated sample and sample treated with GA at different concentrations, respectively, were determined by Student’s t test. ns: no significance; *, *p* < 0.05; **, *p* < 0.01. (**C**) Effect of GA on luciferase expression in pCMV-luc-transfected cells. Monolayers of 293T cells were transfected with pCMV-luc for 16 h. The cells were then treated with GA at indicated concentrations. Luciferase activity was determined after 24 h incubation. GA treatment did not affect luciferase expression delivered by an encoding plasmid. The experiment was performed twice. The readings from untreated samples were used as a control for the calculation of relative luciferase activity. Data are mean ± SD of duplicate wells from 2 independent experiments. (**D**) Time of GA addition on Lenti-S-mediated luciferase gene delivery. Vero E6 cells were untreated or treated with 3 mM GA at 2 h prior to (−2 h), during (0), or at 2 and 4 h post Lenti-S infection. Luciferase activity was determined 24 h PI. The experiment was performed 2 times. Data from the untreated controls were used for the calculation of relative luciferase activity. **, *p* < 0.01.

We used an S protein-pseudotyped lentivirus to determine whether GA had an antiviral effect against SARS-CoV-2. The lentivirus system is easy to construct and has been widely used for virus binding and infection assay. To this end, cells were infected with varying amounts of Lenti-S in the absence or presence of varying amounts of GA (Figure 1A). We tested GA at concentrations of 0.5–5 mM since previous studies showed that GA was previously reported active against a wide range of enveloped viruses at concentrations of 1–8 mM [10,12]. Vero E6 cells in 96-well plates were untreated or treated with GA at 0.5, 1, 2.5, and 5 mM 30 min prior to Lenti-S infection, and we found that GA treatment resulted in a reduction in luciferase activity (Figure 1B). At 2.5 and 5 mM, GA treatment reduced Lenti-S-mediated luciferase gene delivery by approximately 77% and 92%, respectively. The effect of GA on Lenti-S infection was specific since treatment of 293T cells transfected with pLenti-CMV-luc did not affect luciferase expression (Figure 1C).

The time effect of GA addition was tested by treating Vero E6 cells with 3 mM GA at 2 h prior to (−2 h), during (0 h), or at 2 h and 4 h post Lenti-S infection. Luciferase expression was determined at 24 h PI. As shown in Figure 1D, GA addition prior to or during Lenti-S inoculation significantly blocked Lenti-S infection. For comparison, addition of GA at 2 and 4 h post Lenti-S inoculation showed diminished effect against Lenti-S infection. This result suggests that GA likely targeted the early stages of Lenti-S infection.

### 3.2. GA Effect on S Protein Binding to Host Cells

**Figure 2:**
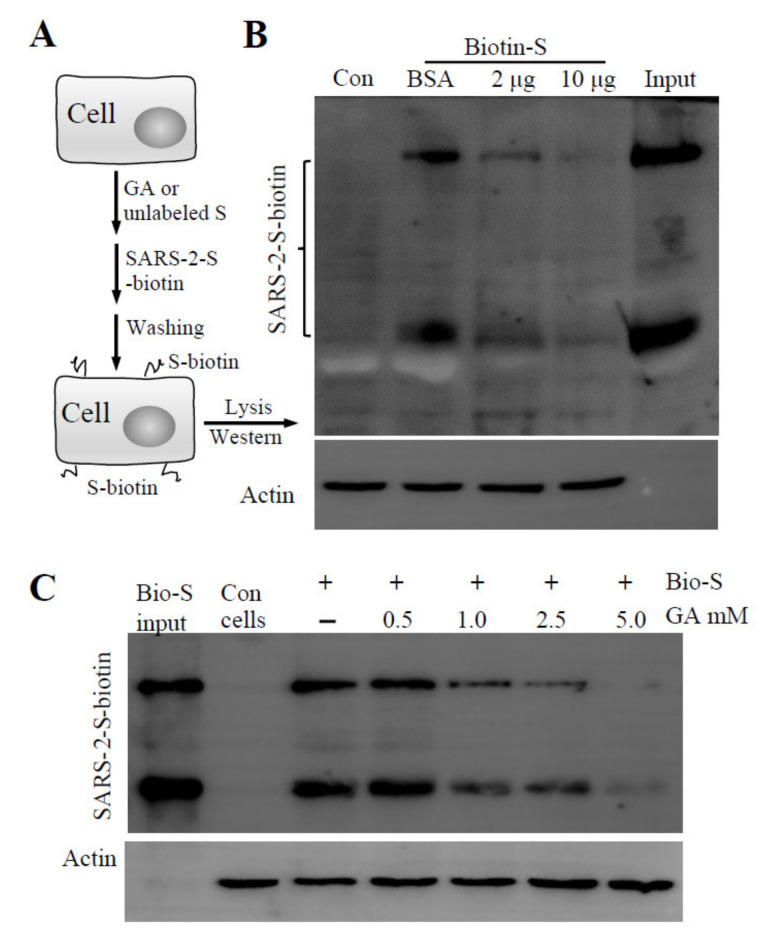
Effect of GA on recombinant S protein binding. (**A**) Schematic presentation of the experimental design. The cells were detached and incubated on ice for 60 min with GA or recombinant S protein (S unlabeled). Biotinylated S protein was then added for cell-attachment assay. After washing with ice-cold DMEM, cell-attached biotinylated S protein was detected by immunoblotting assay. (**B**) Biotinylated S protein binding to host cells in the presence of BSA or unlabeled S protein. Vero E6 cells resuspended in ice-cold DMEM containing 0.5% BSA were allowed to interact with Lenti-S in the presence or absence of unlabeled S protein (at 2 and 10 μg/mL). (**C**) Effect of GA on S protein binding to Vero E6 cells. A biotinylated S protein was allowed to bind to detached Vero E6 cells in the absence or presence of GA. Cell-bound biotinylated S protein was determined by immunoblotting assay. Actin was used as a loading control. Con, Vero E6 cells only. Input, biotinylated S protein for total binding.

Virus infection is initiated by receptor-mediated attachment, followed by cell entry via membrane fusion or endocytosis. Since Lenti-S is a pseudotyped virus that utilizes S protein for cell attachment and cell entry, we focused on whether GA targeted the S protein of SARS-CoV-2 for antiviral action. We performed a cell-binding assay using a biotin-labeled S protein to assess whether GA interfered with S protein attachment to host cells. In this regard, a biotinylated S protein was allowed to bind to Vero E6 cells in the presence or absence of GA (Figure 2A). After washing off non-bound S protein with an ice-cold medium, cell-bound S protein was detected by immunoblotting analysis. We used unlabeled S protein as a competitive reagent against biotin-labeled S protein binding to demonstrate the binding specificity of biotinylated S protein to host cells (Figure 2B). As shown in Figure 2C, GA treatment dose-dependently inhibited S protein binding to the cells. At 5 mM concentration, GA blocked S protein binding by more than 95%, suggesting that GA inhibited pseudovirus infection likely by blocking S protein-mediated cell attachment.

### 3.3. GA Treatment of Pseudovirus but Not the Cells Inhibits Pseudovirus Infection

**Figure 3:**
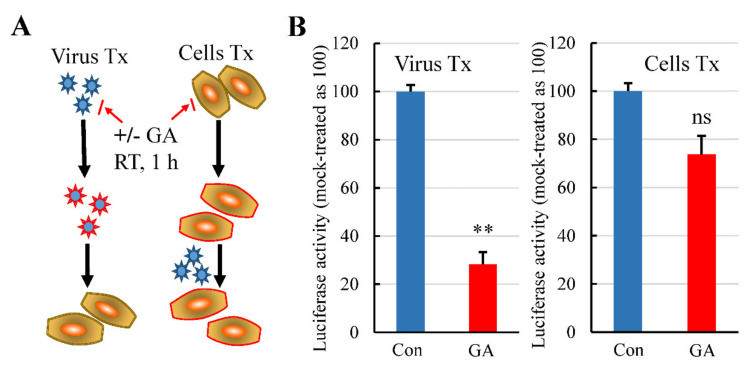
Pretreatment of Lenti-S or host cells with GA on Lenti-S mediated luciferase gene delivery. (**A**) Diagram showing Lenti-S or host cells were treated (Tx) with 3 mM GA prior to the infection assay. Both Lenti-S and host cells were untreated or treated with 3 mM GA for 1 h. At the end, the GA-treated Lenti-S was 1:30 diluted and used to infect untreated host cells (final concentration of GA in the medium was approximately 0.1 mM). In parallel, the medium of the GA-treated cells was replaced with fresh medium without GA followed by infection of untreated Lenti-S. Luciferase activity was determined 24 h later. (**B**) GA effect on Lenti-S (Virus Tx) and on host cells (Cells Tx). Luciferase activity was expressed as a percentage of untreated controls. Data are mean ± SD of duplicate wells from 2 experiments. ns, no significance; **, *p* < 0.01 by Student’s *t* test.

We performed an infection assay by pretreatment of the virus and the cells to preliminarily determine a primary target of the GA effect. In this regard, Lenti-S was pretreated with GA at a concentration of 3 mM on ice for 1 h. The treated virus was then 1:30 diluted with culture medium and used to infect the cells (final GA concentration in the culture medium was at approximately 0.1 mM; the concentration showed no antiviral activity). Virus infection was determined by measuring luciferase activity at 24 h post infection. Alternatively, we also treated the cells with 3 mM GA on ice for 1 h to determine whether GA targeted the host cells for its antiviral effect since the receptor ACE2 was a putative target of GA action [22]. After removal of GA by rinsing with fresh DMEM, the cells were then infected with Lenti-S for 24 h. Pretreatment of cells with GA showed marginal effect against Lenti-S infection, while pretreatment of pseudovirus with GA profoundly reduced pseudovirus infectivity (Figure 3), indicating that GA targeted virus particles for the antiviral effect.

### 3.4. GA Interacts with S Protein

**Figure 4:**
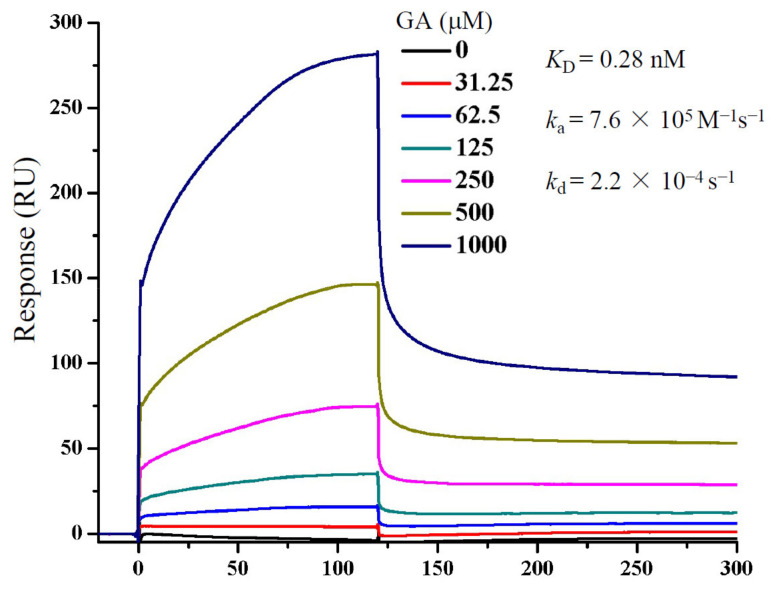
Determination of GA binding to S protein by SPR. S protein was immobilized on to a CM5 sensor chip. GA at concentrations as indicated was passed over the chip, and SPR angle changes were recorded using the Biacore T200 system and reported as response units (RUs). Data fitting was performed using the 1:1 Langmuir model in the BIAevaluation software package (GE Healthcare).

To more clearly demonstrate whether GA interacted with S protein, we performed an SPR assay to measure GA interaction with a recombinant S protein (Figure 4). The *k*_a_ and *k*_d_ were measured at approximately 7.6 × 10^5^ M^−1^s^−1^ and 2.2 × 10^−^^4^ s^−1^, respectively, which translated to a calculated *K*_D_ of 0.28 nM, if a 1:1 stoichiometry was used for GA and S protein interaction. The result shows clearly a direct interaction between GA and S protein, although this might have overestimated the affinity since the S protein is believed to be a homotrimeric protein which would have more than one GA binding site per protein. Regardless, the results together indicate that GA blocked Lenti-S infection through inhibition of S protein-mediated cell binding.

### 3.5. Autodocking Reveals GA-Binding Pockets on SARS-CoV-2 S Protein

**Figure 5:**
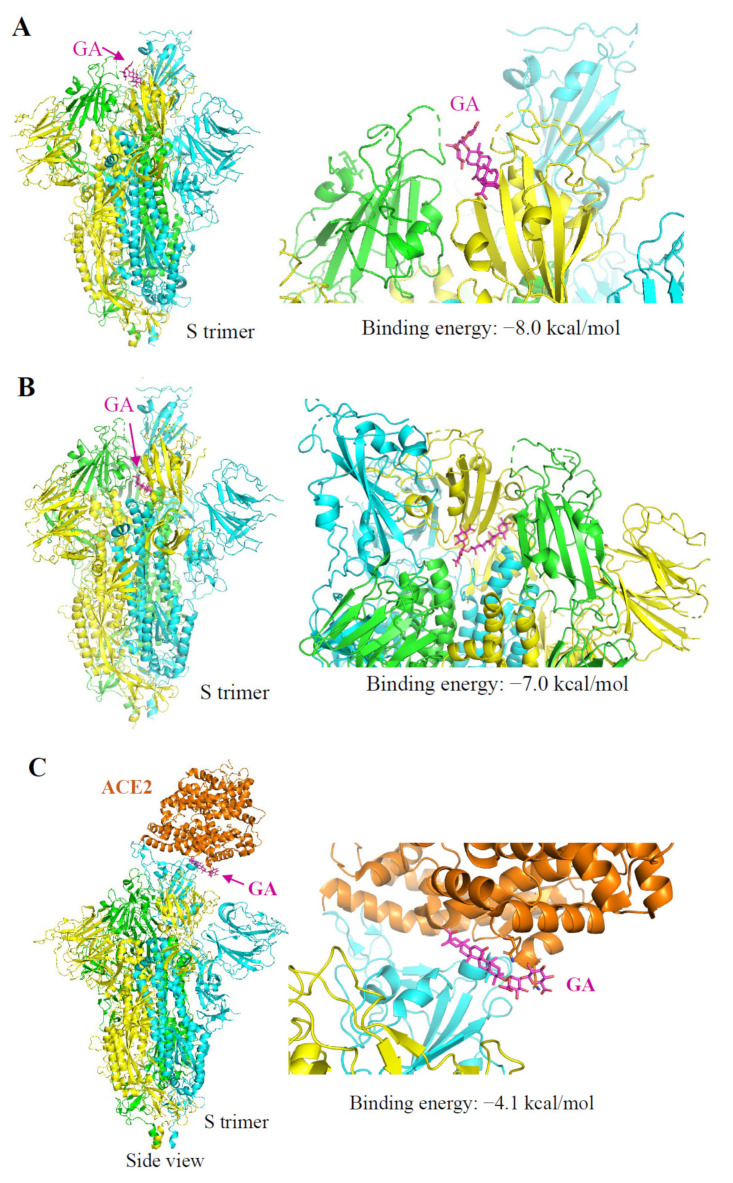
Autodocking analysis of GA interaction with SARS-CoV-2 S and ACE2 protein. Three protomers of SARS-Cov-2 S protein (PDB id: 6vsb) are shown in cyan, green, and yellow, respectively. ACE2 protein (PDB id: 6m18) is in orange. The structure of proteins is presented in ribbons. The structure of GA (ZINC id:960251743495) is shown in magenta as sticks. Arrows indicate the predicted binding site of GA. (**A**) Predicted binding of GA on the S protein at the S–ACE2 interface with a calculated binding energy of –8.0 kcal/mol. (**B**) Predicted binding of GA on a binding pocket located at the inner side of the RBD with a binding energy of −7.0 kcal/mol. (**C**) Predicted binding of GA on the ACE2 protein at the ACE2–S interface with a binding energy of −4.1 kcal/mol.

It was predicted that the RBD of the S protein contains binding pockets for natural products including GA [23]. We performed AutoDock Vina analysis by scanning through the entire extracellular domain of the S protein for the binding potentials. The first notion was GA might bind on the interaction interface of S protein–ACE2 since we found that GA treatment blocked Lenti-S infection and S protein attachment. Indeed, a binding pocket at the S-ACE2 interface was identified with a calculated binding energy of −8.0 kcal/mol (Figure 5A). The S protein presents in two different conformations, named open and closed states [24]. We also identified another binding pocket located at the inner side of the RBD with a binding energy of −7.0 kcal/mol (Figure 5B). SARS-CoV-2 opening is expected to be necessary for interacting with ACE2 at the host-cell surface and initiating the conformational changes leading to cleavage of the S2 site for efficient membrane fusion and viral entry [25]. It is possible that GA binding at this inner binding pocket impacted the close–open state transformation resulting in diminished open-state trimer to interact with ACE2. Thus, we also searched for the potential binding pocket at the ACE2 receptor. Similar to a previous report [22], a pocket at the interface binding to the S protein provided a binding energy of −4.1 kcal/mol (Figure 5C). Based on the predicted binding energy, we speculated that the primary binding site of GA should be on the S protein rather than the ACE2.

In summary, we showed here that GA interacted with SARS-CoV-2 S protein and blocked S protein-mediated cell binding for the antiviral activity of GA against SARS-CoV-2.

## 4. Discussion

Coronaviruses are a group of related viruses that cause diseases in humans and animals. In humans, coronaviruses cause respiratory tract infections, ranging from the common cold to the deadly diseases by SARS-CoV, MERS-CoV, and SARS-CoV-2. Due to the lack of medicines for COVID-19, repurposing currently existing and experimental drugs has been proposed as an alternative to uncover agents with therapeutic potentials. Traditional medicines have demonstrated records as anti-infectives throughout the history of mankind and have shown to be effective in China at alleviating COVID-19 symptoms or even reducing fatality. GA, a major component of *Glycyrrhiza* spp., possesses a wide range of pharmacological and biological activities, including antioxidant, antiviral, and anti-inflammatory effects [7,13,26,27]. Gowda and colleagues reported that GA could inhibit SARS-CoV-2-protein-induced high-mobility group box 1 (HMGB1) release and inhibits viral replication [28]. GA also targets SARS-CoV-2 main protease [29] and blocks proinflammatory response [30]. It is likely that GA can utilize multiple mechanisms against SARS-CoV-2 infection and disease [29,30]. To initiate a productive cycle of infection, a virus first attaches to a host cell, followed by a cell entry and replication process. In this study, we used a pseudotyped lentivirus and showed GA with antiviral activity. We focused on the early stages of virus infection using a pseudotyped virus system. This approach has been successfully used to construct pseudotyped lentiviruses for SARS-CoV, MERS-CoV, and recently SARS-CoV-2 and the corresponding mutants [31,32,33,34]. As a model for highly contagious pathogens, pseudotyped viruses are easy to construct and safe to use. It allows in particular the detailed studies involving virus attachment and virus-cell entry stages.

Several studies have predicted S protein or S–ACE2 interaction as potential targets for GA against SARS-CoV-2 [27,35]. Here we provided experimental evidence demonstrating that GA blocks S protein-mediated cell attachment for its antiviral effect. We found that GA interacted with the S protein with high affinity and blocked a recombinant S protein binding to the host cells. We also executed computational molecular docking to elucidate potential GA binding pockets on S protein. We screened through the entire extracellular domain of S protein by defining multiple grid boxes within this region. In addition to a previously revealed GA binding site at the interaction interface between the RBD and ACE2 protein [36], we also found a binding pocket at the inner side of the RBD. Based on the structural features, we predicted that the binding may have several impacts on the infectious activity of Lenti-S. First, S protein presents in two different conformations including a close state and an open state with one RBD of the trimer flipped out. The switch from close state to open state of S protein was necessary to establish an interaction with the ACE2 receptor. The binding site at the inner face of the RBD could impact this conformation transition. Secondly, it is also likely that GA binding at the inner side of the RBD might interfere with subsequent conformational change during the fusion stage.

In an assimilated SARS-CoV-2-infected mouse model, nanoparticles carrying GA demonstrated therapeutic effects through anti-inflammatory and antioxidant activities [37]. At the intracellular and circulating levels, GA binds to high-mobility group box 1 protein (HMGB1) to provide robust anti-inflammatory and neuroprotection [38,39]. GA attenuates pulmonary hypertension progression and pulmonary vascular remodeling in animal models [40,41,42]. As a hydrophilic compound, GA is not readily absorbed. After oral ingestion, glycyrrhizin is first hydrolyzed to 18 β-glycyrrhetinic acid by intestinal bacteria, which can be absorbed from the gut [43]. The metabolites in circulation, along with GA, can significantly reduce inflammatory cell infiltration and cytokine production during an infection [40,42]. 

Pompei and colleagues reported that GA was effective against a broad range of enveloped viruses [10]. Cinatl et al. showed that GA was effective against SARS-CoV [12,44]. The reported concentrations for GA antiviral effect in cell cultures generally vary between 1 and 5 mM concentrations, at which concentrations GA can form an emulsion or long-lasting foams in an aqueous solution [45,46,47]. It is well known that surfactants were able to inactivate enveloped viruses by causing protein aggregation, disruption of the envelope, or by distorting the shape of virions [48]. At the membrane level, GA induces cholesterol-dependent disorganization of lipid rafts which are important for the entry of coronavirus into cells [49,50]. GA was also reported to modulate the fluidity of the plasma membrane and HIV-1 envelope [45]. The fact that GA directly inactivated enveloped virus particles suggests that GA likely exerts its antiviral activity by destabilizing the envelope. Whether chemicals such as GA use the surfactant activity for their antiviral effect remains to be further studied.

Here we provided experimental and computational simulation data demonstrating that GA potentially targets S protein-mediated cell attachment for its antiviral activity. GA interacted with the S protein with high affinity and blocked recombinant S protein binding to the host cells. Thus, this study uncovered a mechanism by which GA blocks SARS-CoV-2 infection, highlighting the potential of herbal medicine against emerging and reemerging infectious diseases.

## Data Availability

Not applicable.

## Sample Availability

Not available.

## Abbreviations

ACE2	angiotensin-converting enzyme 2
BSA	bovine serum albumin
COVID-19	coronavirus disease 2019
DMEM	Dulbecco’s Modified Eagle Medium
EDTA	ethylenediaminetetraacetic acid
FBS	fetal bovine serum
GA	glycyrrhizic acid
RBD	receptor-binding domain
SARS-CoV-2	severe acute respiratory syndrome coronavirus 2
S protein	spike protein of SARS-CoV-2
